# Solar PV-Battery-Electric Grid-Based Energy System for Residential Applications: System Configuration and Viability

**DOI:** 10.34133/2019/3838603

**Published:** 2019-10-08

**Authors:** V. Bagalini, B. Y. Zhao, R. Z. Wang, U. Desideri

**Affiliations:** ^1^University of Pisa DESTEC, Largo Lucio Lazzarino, Pisa 56122, Italy; ^2^Institute of Refrigeration and Cryogenics, Shanghai Jiao Tong University, Shanghai 200240, China

## Abstract

Distributed renewable energy share increase in electricity generation is creating challenges for the whole power system, due to its intermittent and nonprogrammable nature. Energy storage has the potential to solve those issues although its technical, economic, and environmental impact is up for debate. The paper presents a study about a PV-battery energy storage system installed in a grid-connected residential apartment in the Green Energy Laboratory at Shanghai Jiao Tong University, China. Daily experimental results show how the presence of energy storage reduces the midday feed-in of excess PV power and the evening peak demand, providing benefits to the distribution network in terms of reduced voltage swings and peak load. Considering the Chinese context, an economic analysis is carried out to assess the profitability of residential PV-battery systems, using the net present value as the economic indicator of an 18-year investment in which the battery pack is replaced twice (6 life years). The analysis shows that such system is not economically viable due to a combination of low electricity prices, valuable PV incentives, and high technology costs. However, considering a future scenario of doubled electricity tariff, halved export tariff, and falling technology costs (-66% battery and -17% PV and inverter), PV-battery investment becomes profitable and shows more resilience to future scenarios than PV-only investment.

## 1. Introduction

Global demand for electricity is growing, and it might grow even faster with the picking up of the electrification of transport (e.g., electric vehicles) and heat (e.g., heat pumps) [1]. At a geopolitical level, frequent energy crisis and fossil fuel dependency are other crucial factors in shaping the future of the energy sector [2]. One of the most promising solutions to those challenges is the large scale-deployment of Renewable Energy Sources (RES) [3]. Among the RES, wind and solar photovoltaic (PV) are the most interesting due to their potential and their availability pretty much everywhere in the world [4]. However, RES have their issues. From an energy perspective, the main issues are as follows: (1) intermittency, (2) availability, which is not constant in time and space, and (3) nonprogrammability, since their output is weather dependent and cannot be planned, although it can be forecasted [5]. From an economic point of view, they have to face cost competition from traditional sources of electricity generation, such as fossil fuel-fired power plants [6]. Electrical Energy Storage (EES) is being put forward as a potential solution to RES energy issues, mitigating the impact of shifting from traditional sources of electricity production and paving the way to a substantial increase in RES penetration into the overall production share [7]. The popularity of energy storage at a residential level is growing due to its potential positive contribution to the technical operation of the electricity distribution network and to the economic performance of PV systems in future scenarios [8]. The advantages of storage for the distribution network are paramount. Storage is starting to be recognized as an important player by regulators and operators resulting in the appearance of EES-tailored incentive schemes [9] and the installation of utility-scale systems serving different purposes [10]. Considering that small residential PV plants have had a huge impact on the overall network, deploying storage at the same level should have similar effectiveness [11]. In the meantime, from an end user point of view, the installation of storage must have some positive economic impacts. The main operating strategies of profitable commercial and residential energy storage are the following: (a) load shifting/peak smoothing operations, where profit comes in the case of time-of-use tariffs, charging the battery at low price from the grid and discharging it at high price to meet the local demand [12], and (b) self-consumption of associated PV system improvement, where profit comes from the difference between the electricity import tariff and the selling price [13].

This work focuses on battery systems associated with PV generation plants at a residential level, with the purpose of maximizing self-consumption [14–16]. This operation strategy naturally smoothes the interaction of a residential PV system with the grid, and, due to the nature of PV production (daytime peak) and domestic load profile (evening peak), it also results in an indirect overall load shifting effect. As far as PV generation is concerned, seeking help and synergy from storage are a popular topic in the academic and commercial world. The influence of storage devices on the profitability of residential PV projects has been assessed in many studies [17] with controversial results, although they all convey in defining some conditions that would make the overall investment attractive. Germany and Italy, due to the high electricity prices at times of decreasing PV module cost, became among the first countries to reach grid parity. The installation of PV-battery systems in private households is aimed at increasing self-consumption of the PV energy and thereby the home owner's self-sufficiency. Now that PV generation incentives are being phased out and the amount of energy that can be fed into the grid is being limited, PV-battery systems are expected to be profitable in a few years even without incentives due to decreasing investment costs [18]. An Italian study concludes that energy storage associated with the PV system is useful only when the relationship between supply and demand permits them to induce a significant increase in energy self-consumption [19]. Another study focusing on the Italian electricity sector concludes that PV-battery storage systems are economically unsustainable compared to PV-only systems harnessing the net metering scheme. Even without this PV incentive scheme, the installation price for energy storage would need to come down considerably to make the addition of storage convenient [20]. Similarly, a Portuguese study concludes that self-consumption is already attractive, but storage is not a profitable solution, because battery investment is still too high, despite the cost reduction witnessed in recent years [21]. A Germany-based study found that already in 2013, small-sized PV-battery storage systems were economically viable without premium payment for PV generation or incentive for self-consumption, while higher electricity retail prices and lower electricity wholesale prices added profitability to such systems, increasing also battery capacity and PV sizes of the optimum configuration [22]. In the UK, the PV-battery storage system in the commercial sector has been evaluated both economically and environmentally with the conclusion that PV would be economically attractive on its own by 2020 even without incentives. Adding a battery would improve the overall economic performance only if costs came down enough [23]. One of the latest UK studies on the profitability of domestic PV-battery systems criticizes all previous studies for neglecting battery degradation effect properly and concludes that, on top of the system not being profitable under current circumstances, adding the consideration of battery degradation worsens the outcome significantly [24].

All those studies have different final outcomes but are clearly on the same page in terms of identifying the major factors affecting PV-battery storage system profitability, with cost of batteries being the major one, followed by electricity tariffs and incentives. Mismatch between the load profile and PV generation is also considered the main reason why storage could make a positive impact on such PV-battery systems whose purpose is to increase self-consumption. By studying the PV-battery storage system technical and economic performance in the Chinese electricity context, this work is aimed at additionally contributing to the controversial topic of economic profitability of the PV-battery storage system in the residential sector. The scope of this work is the application of a battery energy storage system (BESS) coupled with PV generation to a residential electricity user connected to the low-voltage distribution network in Shanghai, China.

## 2. Experimental and Simulation Results and Economic Analysis

### 2.1. Experimental Results on Typical Summer Weekdays

The PV-battery system (see Methods) behavior has been monitored under different weather and load conditions, throughout the summer of 2018. All data have been collected experimentally by the inverter, elaborated, and analyzed with the purpose of getting an insight into the system operation. Power data was recorded at a one-minute interval. The electrical demand profile represents the potential domestic usage of a couple or small family. The members of this family are assumed to be working out of the house during weekday's office hours whilst staying at home during evenings and weekends. Two students acted as dwellers and moved to the apartment every evening at around 6 p.m. to make use of the electrical appliances as if people were living there. Air conditioning and lights were turned on as required, meals were cooked for themselves and guests, laptops were plugged in, phones were charged, TV was switched on, and also other appliances were occasionally used. At night, the air conditioner would be kept on or off depending on the actual need of it.

**Figure 1:**
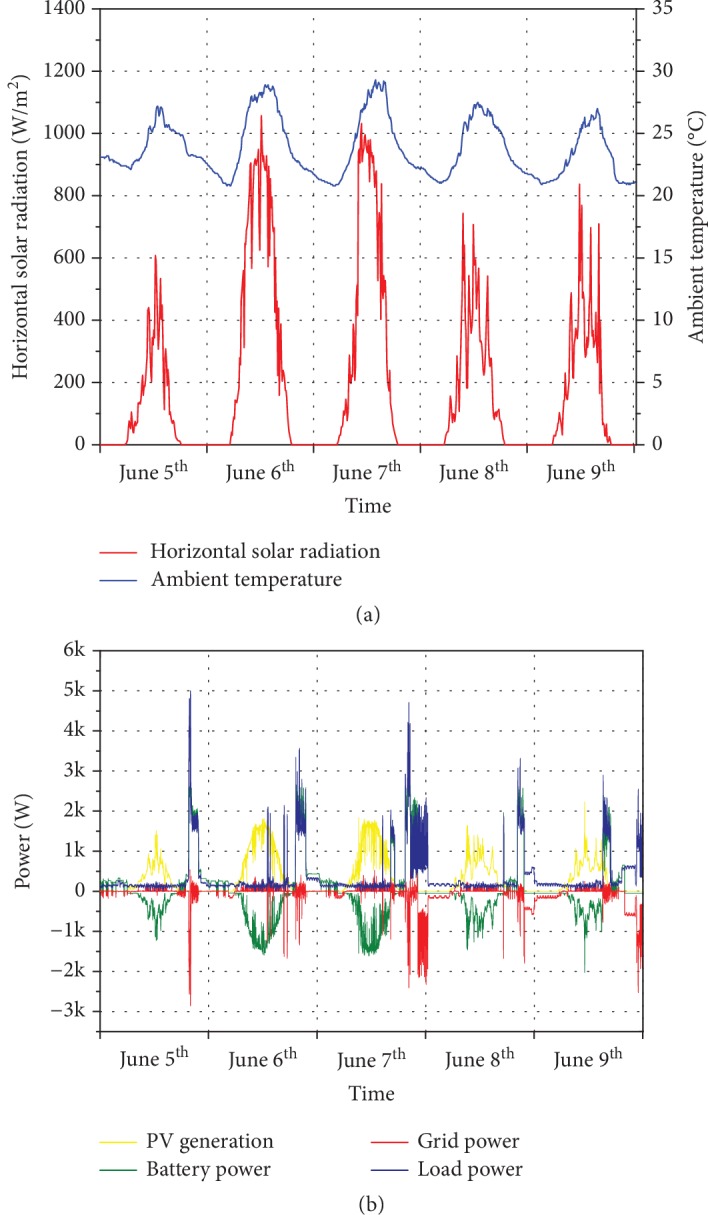
Monitored data from 5^th^ to 9^th^ of June 2018: (a) weather parameters including ambient temperature and solar radiation; (b) measured values of main energy flows.

Results shown below represent the behavior of the system for 5 consecutive weekdays, say Monday to Friday, of the summer season. Inverter data were recorded from the 5^th^ to the 9^th^ of June 2018 with temperature ranging between 20°C and 30°C and fairly variable weather with some rainy days limiting the PV yield. The recorded weather data for the chosen period is shown in Figure 1(a). All the main energy flows are shown in Figure 1(b). While PV was producing excess energy, the battery was charging (negative values); then, when the load kicked in, the battery was discharged (positive values). Grid import is represented by negative values, and it occurred only when neither PV production nor battery discharging could meet the local demand.

**Figure 2:**
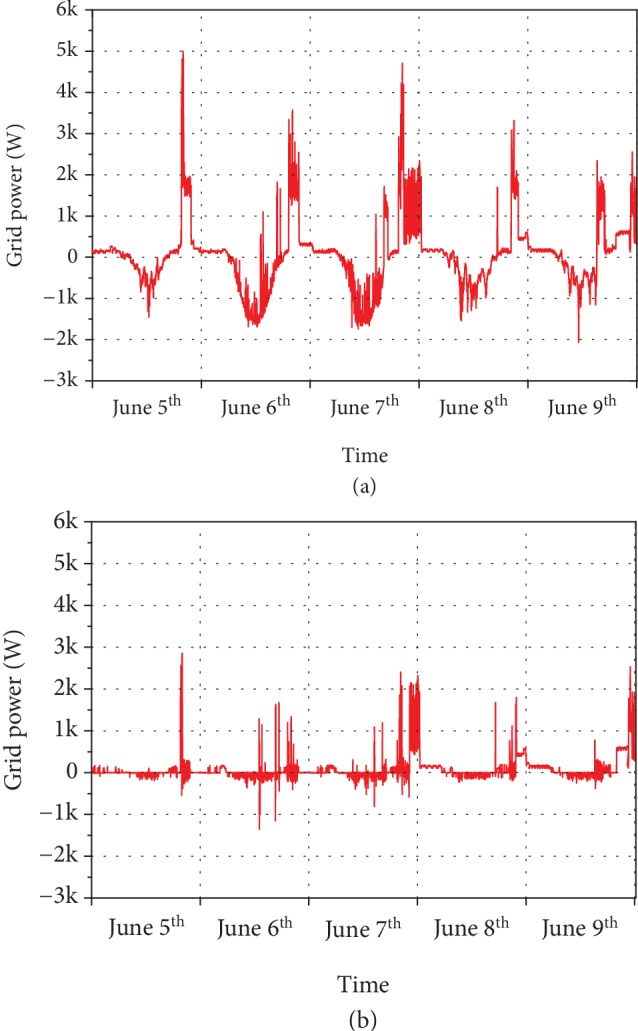
Grid export (negative values) and import (positive values) (a) in the absence of storage and (b) in the presence of storage.

Before showing the actual behavior of our system in terms of battery operations and energy flows to and from the grid, it is interesting to notice that in the absence of energy storage, the grid would have experienced an energy flow given by the combination of demand and PV production.  Figure 2(a) shows how the alternation of daytime PV production peaks (-2 kW) and evening consumption peaks (up to 5 kW) could potentially stress the network with a net 7 kW of power swing in 6 hours between midday and 6 p.m. Adding storage changes the situation as expected with reduced peaks both in generation and in consumption modes, as shown in Figure 2(b). The amount of energy bought from the grid at peak time 6 p.m.-10 p.m. is also reduced. This is a great example of how the PV-battery system strategy of maximizing self-consumption has the precious side effect of peak shaving.

**Figure 3:**
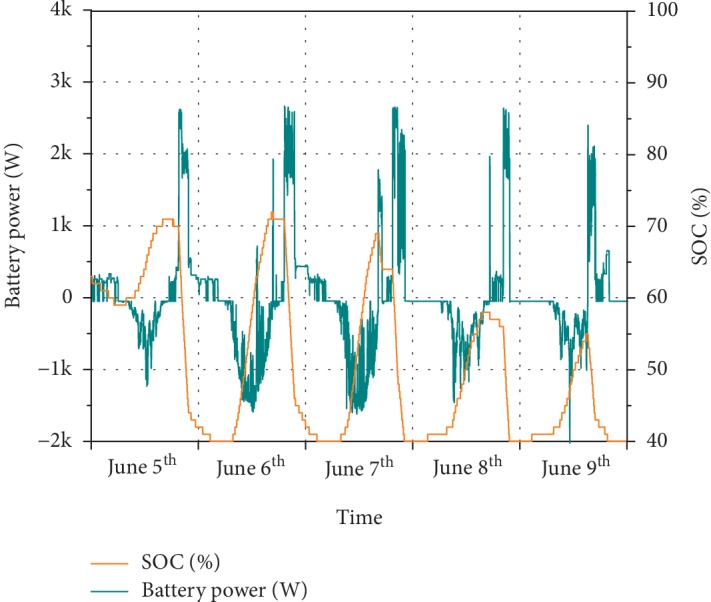
Battery SOC in % on the left axis. Battery power on the right, positive when discharging and negative when charging.

The operation of the battery is highlighted in Figure 3. Battery power was limited to 2.5 kW both in charging and in discharging modes to limit battery ageing. DOD (Depth of Discharge) was also limited to 60%; hence, the SOC (state of charge) ranged between 40% and 100%. It can be noticed from the SOC graph that the battery was never brought above 72% SOC. This is due to the relatively big size of the battery (14.4 kWh) compared to the PV generation size (3 kWp nominal, around 2 kWp in practice). The system was not designed for the specific application described in this work, so it is far from being optimally designed. The topic of optimal sizing of this PV-battery storage system is presented in Methods.

Considering the initial and final SOC for the chosen period of 63% and 40%, corresponding to 5.52 kWh (referred to as ΔSOC) of additional available discharge energy, an average battery round-trip efficiency for this specific period could be calculated as

(1) $$\begin{array}{c} \text{Average round}‐\text{trip efficiency}=\frac{\text{Energy discharged}}{\text{Energy charged}+\Delta \text{SOC}}=\frac{28.34\;\left(\text{kWh}\right)}{28.5+5.52\;\left(\text{kWh}\right)}=0.83. \end{array}$$

This needs to be taken carefully as the performance of the battery depends on its usage. However, as far as typical summer weekdays are concerned, this value can be taken as a reference.

Based on this 5-day operation, it can be seen how 44.05 kWh of demand has been satisfied mainly by self-consumed energy, with only 11.64 kWh of the energy bought from the grid. Of the remaining 32.41 kWh, 28.34 kWh must have come from the battery discharging although some of this has been wasted through the conversion operation. PV production did mainly charge the battery, while only 2 kWh has been sold and the rest has been consumed locally at the time of production. Those are just example values from a 5-summer-weekday operation, which favors storage due to higher PV production coupled with evening loads.

### 2.2. Simulation Results of the PV-Battery System Compared to the PV-Only Benchmark

In this section, annual simulations (see Methods) are run to assess the energy performance of the system's overall load and weather conditions, with weather data of the typical meteorological year type, TMY2. The model is set up to represent the real system installed at the Shanghai Jiao Tong University Green Energy Lab (GEL), as per the validation simulations: 3 kWp of PV generation and 14.4 kWh of usable battery capacity.

The demand is composed of a thermally modelled HVAC part (see Methods), while the rest of the electricity load is imputed as a daily routine assuming a fixed schedule for some of the most commonly used electricity loads (see Table 1). The assumption is that the apartment's dwellers, a couple or small family, are not at home during office hours in weekdays (from 8 a.m. to 6 p.m.) while they are at home for the rest of the time, that is, weekday evenings until the following morning and weekends. This will affect the working hours of the air conditioning system and the other electrical load, with the HVAC system switched on every evening at 6 p.m. during weekdays, until 8 a.m. the following day, while during weekends, it remains on all the time. The indoor temperature set point is 20°C in heating mode and 25°C in cooling mode.

To have a quantitative assessment of the operation of the system in both configurations with and without energy storage, all yearly energy flows are reported in Table 2 (actual residential). Some performance indicators have been included. The self-consumption rate (SCR) represents the amount of locally produced renewable energy that is consumed on site and not sold to the grid. The self-sufficiency rate (SSR) represents the amount of load energy that is met by locally produced renewable energy. The contemporaneity of production and consumption plays a big role in systems without energy storage. Smaller PV plants compared to the local demand are likely to have higher self-consumption rates as a bigger share of their production can be locally consumed. Storage provides a means to “artificially” increase self-consumption by storing the excess energy and using it at a later time. Self-sufficiency is a different concept about independency from the grid. It is intended here as the self-consumed energy over the total demand. Self-production rate is more about the net energy balance. It is simply the ratio between energy produced locally by the PV plant and the total demand, without considering the amount of self-consumption. The addition of battery energy storage to the PV system increases both self-consumption and self-sufficiency, although in general the latter is more strongly affected by the system's local demand.

By comparing the energy behavior of PV-battery and PV-only systems, it is found that the presence of the battery reduces peak power to and from the grid. For the system with actual sizes (residential SH), the energy sold to the grid is almost eliminated and there is a 60% reduction in the amount of electricity bought. Furthermore, while the share of self-produced energy does not change, more of this locally produced energy is used on site bringing the SCR from 24% to 79% and the SSR from 20% to 68% (see Table 2).

### 2.3. Optimum System Sizing

The actual sizes of the real system components installed at SJTU GEL are 3 kW PV peak generation power and 24 kWh gross/14.4 kWh usable battery energy capacity. Those values have not come out from a proper sizing operation tailored to this type of system, load profile, and operating mode. Consequently, as discussed in the experiments and full-year simulation utilizing these sizes, the battery does not utilize all its usable battery capacity, most of the time either failing to charge up to 100% SOC in winter or working at high SOC in spring.

Finding the optimum sizing is an operation to be carried out at the design stage, once technologies for storage and PV, operation strategy, load profile, and economic parameters are given. In this case, by keeping the same technologies, operating strategy, load profile, and economic parameters, a better sizing is found according to [25], where it is concluded that for a residential PV-battery system aimed at increasing self-consumption in a future scenario of decreasing technology costs and falling PV generation incentives, the optimum PV peak power and battery usable capacity sizes normalized to the total yearly user electricity consumption (MWh_yc_) are around 0.8 kW/MWh_yc_ and 1.1 kWh/MWh_yc_, respectively. In this case, MWh_yc_ is equal to 4.3, so the PV size would be 3.5 kWp and the battery pack would have a usable capacity of 4.75 kWh, corresponding to around 8 kWh of total capacity considering a maximum of 60% DOD during its utilization. Full-year simulation of the system using the model with different PV and battery sizes is carried out. Energy flows and economic results are shown in Table 2 (optimum residential) for both PV-battery and PV-only cases, for systems with actual and optimum sizes.

Furthermore, in order to evaluate how the load profile influences the system performance, the simulation of the system with a nonresidential load profile [26] using the optimum sizes was conducted and the results are shown in Table 2 (optimum nonresidential). The nonresidential load profile refers to office-type building scenarios where the main load occurs in the day time, while the residential load represents a pattern of a household dwelled by people working a normal 9-5 job, showing an “evening power peak.” Meanwhile, to investigate the influence of climates, we presented the simulation results in Haikou (HK, in southern China) with the same system configuration as that in Shanghai. Haikou has a tropical monsoon climate where there are extreme heat events which are not common in Shanghai. The PV incentives, electricity cost, and calculation of NPV in Table 2 can be found in Methods.

From these results, it can be concluded that the “actual size” PV-battery system is not properly designed, mainly resulting in storage being underutilized increasing the capital cost of the system without obtaining enough benefits. The resulting large negative end of investment NPV is the confirmation. With the new proposed “optimum” sizes, which are slightly bigger in the PV plant and much smaller in the battery storage pack, much better results are obtained. However, the NVP value at 18 years is still negative, making the investment unworthy, especially compared to the photovoltaic investment on its own that has positive NPV. This is an expected result as the cost of the batteries still increases the capital investment burden without providing substantial economic benefit in the presence of policies aimed at incentivizing PV production without focus on self-consumption and in a context of cheap electricity tariff. Reduction in technology cost is the other factor having great influence on the financial analysis. When it comes to the load profile, the nonresidential scenario with a PV-only setting exhibits a better consistency between the PV generation and building load than the residential one, inferring from its higher SCR and SSR, and thus, a higher NPV is observed for the nonresidential load profile. However, with the integration of batteries, there is little difference in NPV between the residential and nonresidential cases. In both cases, the battery helps to increase the SCR and SSR of the system, but the battery integration seems more favorable in the residential case since a larger improvement of SCR and SSR is observed, implying a better potential of such system applied in residential buildings. As for the local climate, the optimum sizes still fit the case in Haikou because there is little difference between the total yearly user electricity demands (the basis to determine the sizes of PV and battery capacities [25]) of the two cities. However, the NPVs of both PV-battery and PV-only systems in Haikou are lower than those in Shanghai. The reduction of the NPV is attributed to the decline of PV generation in Haikou instead of the regional temperature difference since these two cities share similar improvements of SCR and SSR after the battery is integrated. The climatic difference does not lead to obvious changes of energy and economic characteristics of such systems.

### 2.4. Sensitivity Analysis on Economic Parameters

PV-battery storage systems, operated to increase self-consumption, in the Chinese residential sector are not economically viable in the current context of low electricity tariffs and valuable PV generation incentives, despite clearly having the potential to solve crucial issues and offer wider technical benefits to the power distribution network. However, there are many economic parameters playing decisive roles in the outcome of such economic assessments, and they are likely to change in the near future. Electricity tariffs, for example, are rising. Incentives for PV generation are decreasing or could be tailored to promote energy storage. Last but not least, the massive interest in electrochemical rechargeable batteries in energy storage and other applications, such as EVs, is increasing sector R&D and product manufacturing volumes, hence improving performance and driving costs down. All these effects when put together not only can but also most likely will change the economic viability of residential energy storage systems.

Sensitivity analysis on different parameters is carried out considering the energy flow simulated by the model with “optimum” sizes of 3.5 kWp PV power and 8 kWh battery capacity, to find out which parameters or combinations of them have most influence on the profitability of PV-battery systems (note: The variation of such parameters does not happen year on year within a given NPV analysis, but those parameters are kept constant for the whole investment period. The variation is considered from one full NPV analysis to another).

#### 2.4.1. Sensitivity Analysis on Electricity Import and Export Tariffs

In this work, the focus is on the coupling of PV generation and battery storage system with the aim of maximizing self-consumption, meaning that less energy will be both sold to and bought from the grid, so increasing the difference between buying (import tariff: expected to grow) and selling (export tariff: could be lowered or removed) electricity tariffs is expected to improve PV-battery storage systems' economic performance compared to that of PV-only systems.

**Figure 4:**
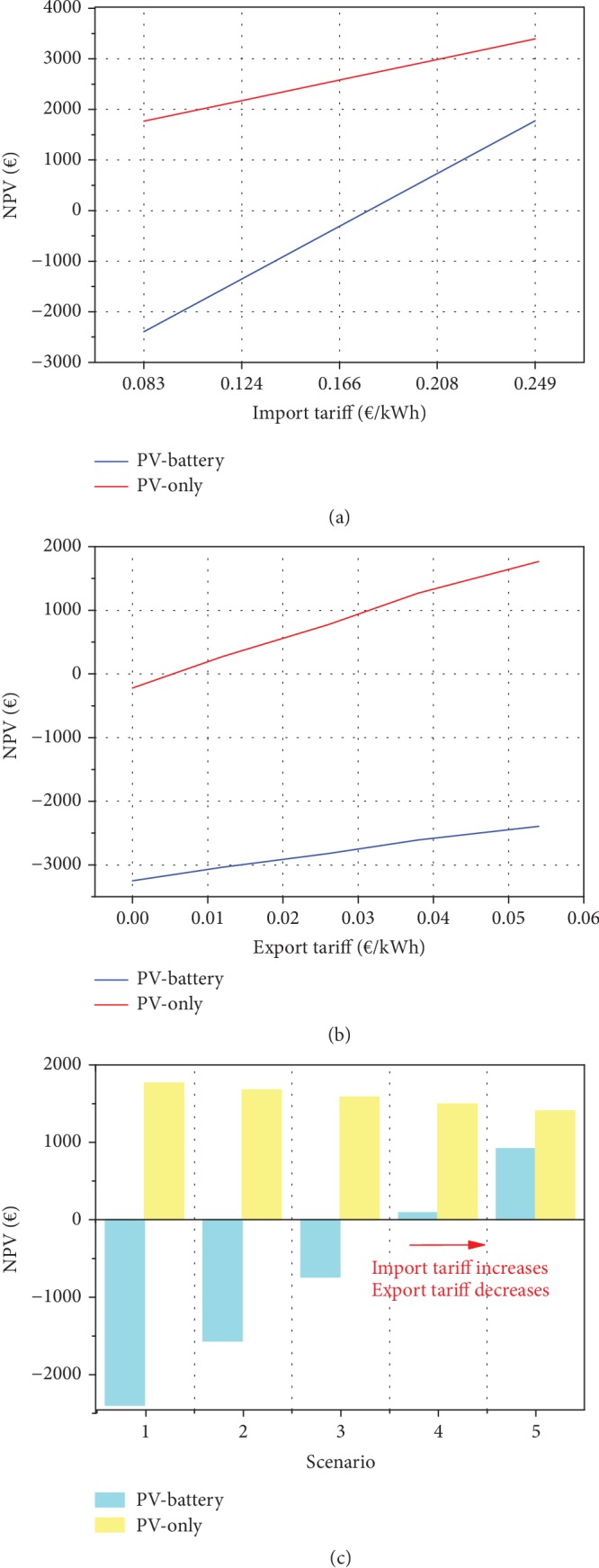
NPV trend with varying (a) import tariffs at a constant export tariff and (b) export tariffs at a constant import tariff and in (c) different scenarios (see Table 4).

Current peak electricity tariff is 0.083€/kWh, and export tariff is 0.054€/kWh. In this sensitivity analysis, the electricity tariff is considered to be flat, so no time-of-use tariff is considered. The rest of the parameters are kept as they are in the base case; e.g., PV incentives apply as per previous analysis. At first, only the import electricity tariff is increased, keeping the export tariff constant; then, the opposite applies. It can be noticed from Figure 4(a) that increasing the electricity tariff increases the NPV of both PV-only and PV-battery systems, but the latter curve is steeper. Likewise, decreasing the export tariff (see Figure 4(b)) has a negative effect on both systems, but the PV-battery system is less affected. The combined effect of falling export prices for excess PV generation sold to the grid and rising import electricity tariffs rewards the PV-battery system.  Table 3 shows 5 scenarios in which the import electricity tariff is gradually increased, while the export one is gradually decreased. From Figure 4(c), it can be found that in case of tripling import tariff and elimination of export tariff, the PV-battery system achieves economic viability (positive NPV) and almost reaches PV-only performance. However, the battery is still underperforming compared to the PV-only scenario. It should be kept in mind that the previous analysis was carried out with PV incentives in place; the next section will focus on studying their effect.

#### 2.4.2. Sensitivity Analysis on Incentives

In Shanghai, there is a double reward for energy produced by residential PV systems: a 20-year state subsidy of 0.056€/kWh and a 5-year local subsidy of 0.053€/kWh. The effect of reducing those incentives will be studied first, and then, some incentives targeting self-consumption solely will be tested.  Table 4 shows how the NPV varies for both PV-battery and PV-only systems while incentives are lowered down to zero. Economic performances of both systems are equally affected in similar measure, showing that this does not promote the addition of battery storage systems to PV plants.

One solution could be the introduction of incentive schemes rewarding self-consumption solely. They are incentives on the PV-generated energy that are paid only if the energy is consumed locally by the user, and not sold to the grid. Storing the PV-produced energy in the battery equals to self-consuming it as it is not sold, but used at a later stage. In this scenario, the export tariff on the excess energy sold to the grid is still in place. The user in fact would still earn money for the energy sold to the grid, but it would lose the generation incentive on that energy.  Table 5 shows 5 scenarios where “self-consumption-only” PV generation incentives are increased. It confirms that to make PV-battery investment profitable and even preferred to the PV-only option, incentives rewarding self-consumption should be put in place and the value per unit energy should be tripled.

#### 2.4.3. Sensitivity Analysis on Technology Cost

Batteries are being mass produced due to the rising demand in different sectors such as energy storage and EVs, as it has happened with unitary cost of PV modules, which came down vertiginously from the beginning of the renewable energy era in the mid-2000s. While PV modules are expected to become cheaper even further, batteries have obviously a lot more margin. The reduction rate of battery and PV cost is shown in Table 6. At first, only the battery cost reduction is considered (result in the 3^rd^ column); then also, the reduction in PV and inverter costs is considered (result in the last 2 columns).

The results show that while a reduction in battery cost of two-thirds would make the investment profitable (NPV = 54€), not even an 80% reduction (NPV = 543€) would challenge the PV-only investment (NPV = 1405€, as previously shown). Finally, consider that falling costs of all components would benefit both analyses with the PV-only scenario being still the best option, but with PV-battery scenario closing the gap, showing a better trend.

#### 2.4.4. Sensitivity Analysis on Carbon Price

Carbon emission trading is growing vigorously worldwide as an effective measure to motivate the deployment of renewable energy systems. The Chinese government launched its development plan for a national emissions trading system (ETS) in late 2017, and it could take until at least 2020 before the national ETS is fully functional. The PV-battery system is expected to gain more economic benefits by carbon emission trading in the future with the estimated carbon price being larger than 20€/ton CO_2_ after 2025 [27]. The results of the effect of carbon price on the system NPV are shown in Table 7 where the emission factor of CO_2_ for the Chinese grid is 0.76 kg CO_2_/kWh [28].

The results show that the increasing carbon price could effectively improve the NPVs of both PV-battery and PV-only systems. However, similar to PV incentives, the economic performances of both systems are equally affected in similar measure since the revenue from carbon trading is only relevant to the PV production but not to the addition of battery storage systems. The increase in carbon price does not bring significant impulses for battery installations.

#### 2.4.5. Combination of the Above Trends

Either the sensitivity studies above are following forecasts that are most likely going towards the right direction, such as a decrease in technology cost and an increase in electricity tariffs, or they are inspired by possible policies that could be implemented to promote a further PV generation growth but only if storage is aided, so as to lower its negative impact on the grid and offer the possibility to play a positive role in future networks. It is not unlikely that those trends will manifest themselves simultaneously.

Three scenarios have been developed to represent possible future conditions in which PV-storage investments could take place (see Table 8). Starting from the current Chinese electricity tariffs and incentives and current technology cost (current scenario), a mild scenario is obtained by considering import electricity tariff flat and equal to peak tariff increase by 50%, export tariff is halved, PV generation incentives are halved, technology costs are lowered, and carbon price is increased. The medium and strong scenarios are following similar trends of increasing import tariffs and carbon price and decreasing export tariffs and technology cost with the major difference being the nature of the PV incentives, which reward self-consumption solely.

**Figure 5:**
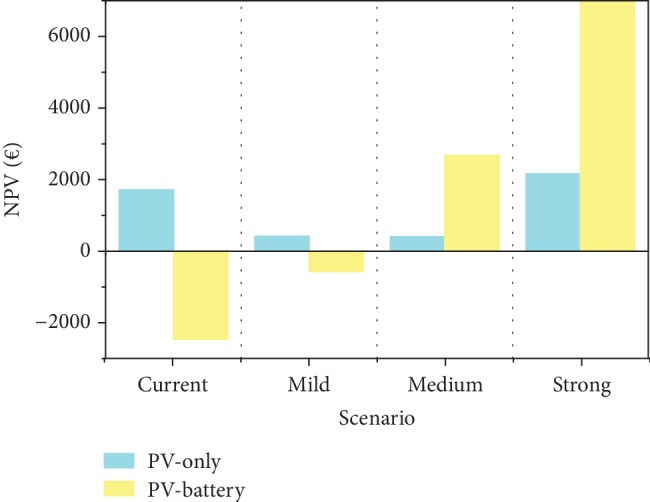
NPV under different future tariff and policy scenarios (see Table 8).

From Table 8 and Figure 5, it can be seen how in the current scenario the PV-only option is profitable while PV-battery is a noneconomically viable investment. Moving on with the scenarios, PV-battery systems become more and more valuable investments with the game changer being the switch from PV generation subsidies (paid to all PV-produced energies) to self-consumed subsidies (paid only to the locally consumed share of PV-generated energy) happening in the medium scenario. The high NPV value of the strong scenario shows how this type of investment has great potential if favorable conditions are met. PV only is not completely compromised though: in the mild and medium scenarios, it is at the edge of profitability, while it is still a valuable investment in the strong scenarios, although whoever will have the capital to add a battery can achieve higher profits.

#### 2.4.6. Optimum System Sizing in Different Scenarios

In the above sections, the sensitivity studies on different important factors in the profitability of the PV-battery system have been conducted based on the assumption of an unchanged “optimum” system size which is discussed in Section 2.3. However, the “optimum” sizing is made under a single scenario (Chinese energy policy and costs from manufacturers in China) and may not fit in other cases. To provide a broader framework to this study, the investigation of optimum system sizing in different scenarios is conducted in this section wherein two assumptions have been made: (1) energy tariffs changed and technology costs unchanged and (2) energy tariffs unchanged and technology costs changed. The objective of the optimization is to maximize the system NPV, and the optimization was conducted by using the TRNSYS-based tool “GenOpt” [29]. In addition, the incentive for PV generation is phasing out in China where the incentive declines from 0.42¥/kWh to 0.18¥/kWh for PV systems built after July 1^st^ of 2019 [30]. It is expected that the incentive would be removed in the near future, and thus, the incentives for PV generation are not considered in this section, which also matches the current situation in Europe.

For the first assumption, we assumed technology costs to be constant (PV system: 1334€/kW; battery: 220€/kWh) and optimized the system sizes under increasing imported tariff and decreasing exported tariff, as shown in Table 9. Under the current imported tariff (0.083€/kWh), the investment of the PV-battery system is not economically viable no matter how the exported tariff changes. When the imported tariff goes up to 0.286€/kWh, the PV-battery system becomes profitable, which accords with some profitable cases in Europe (see Table 10), but the system size is small which indicates that the system does not bring significant economic improvement. With a further increase in the imported tariff, the NPV of the PV-battery system rises notably. On the other hand, the decrease in the exported tariff will cause a slight fall of the NPV. In addition, the increase in the imported tariff will stimulate the rising of both PV and battery sizes, while the decrease in the exported tariff can only reduce the size of the PV system but has little effect on the battery sizing.

From the above analysis, it is found that only when the imported tariff reaches 0.286€/kWh can the PV-battery system become profitable. Therefore, for the second assumption, we assumed energy tariffs to be constant (imported tariff: 0.286€/kWh; exported tariff: 0.054€/kWh) and optimized the system sizes under decreasing PV and battery costs (note: we have tried to optimize the system size under the current imported tariff, 0.083€/kWh, but the NPV remains negative even with the decrease in technology costs), as shown in Table 10. In addition, we set the limit of the size of the PV system at 10 kW in the case that the optimum PV capacity becomes infinite when the marginal cost of the PV system is negative. It is observed that the optimum sizes of both the PV and battery and the system NPV increase as the battery cost declines. However, when the battery cost is lower than 73€/kWh, the increase in PV size becomes less obvious. The decrease in PV cost leads to similar trends of the changes of the optimum system sizes as the battery cost declines, despite the fact that the optimum battery size has little variations when the battery cost is lower than 73€/kWh.

## 3. Conclusions and Future Prospects

This work focuses on grid-connected residential PV-battery storage systems, operated with the purpose of maximizing energy self-consumption. A real system comprising 3 kWp monocrystalline PV modules and 24 kWh advanced lead-acid battery pack (14.4 kWh usable capacity), associated with a grid-connected residential apartment, has been installed at the Green Energy Laboratory of Shanghai Jiao Tong University. The operations of the system have been studied by analyzing experimental data over limited timescales of days, which have been also used to validate a computational model built using the software TRNSYS. The model was used to assess the operations of the system over a full year, giving the possibility to assess its overall energetic performances. Furthermore, the model allowed running additional simulations with different design parameters, like PV power and battery capacity sizes. An “optimum” configuration of 3.5 kWp of PV and 8 kWh of battery capacity (4.8 kWh usable capacity) has been chosen to carry out experiments to be used in the economic analysis.

In respect of energy performance, it is shown that adding battery energy storage to a domestic PV system associated with an evening-oriented electricity demand would reduce the stress of distributed renewables on the grid by limiting the daily exported power. Additionally, the evening peak demand is also reduced. The reduction in power swings from exporting to importing would lower the difference in power requirements during day and night facilitating balancing operation at higher voltages [31]. From full-year simulations of PV-battery energy storage systems whose size has been optimized according to the load, it can be seen how the amount of self-consumed energy increased from 24% to 79%, the amount of purchased energy decreased by 60%, and the amount of energy sold to the grid decreased by 57% compared to the PV only scenario.

In respect of economic performance, PV-battery storage systems in the Chinese residential sector are not economically viable in the current context of low electricity tariffs and considerable PV generation incentives which do not take into account storage. However, considering future scenarios of increasing electricity prices and carbon price, decreasing or self-consumption favorable PV generation incentives, and falling technology costs, the economic outlook of PV-battery investments improves. In particular, it is found that doubling the electricity price would make the PV-battery investment profitable. While lowering the export price shows how the PV-only system is less resilient than the PV-battery system. A combined effect of rising electricity prices and falling export prices would reduce the gap in economic performance between those configurations, although it will not be enough to make PV-battery a better investment than PV only. The sudden removal of PV generation incentives in this context would make both investments unworthy. Instead, one of the most effective ways to promote a battery energy storage system in conjunction with PV generation plants is to introduce incentives rewarding only that part of PV-generated electricity that is self-consumed. Another important finding is that the falling battery cost alone is not enough to make PV-battery system preferable over the PV-only case if all other conditions remain the same. This might mean that it is not enough to wait for battery prices to go down if there is the will to push storage significantly. The economic performances of both systems are equally affected in similar measure by increasing carbon price. Finally, 3 scenarios considering all the abovementioned effects simultaneously have been tested with the result that for the medium scenario, a combination of doubling electricity tariff, reduction to 25% of current export tariff, PV incentives maintaining the current level but rewarding self-consumption only and technology cost reduction (-66% for battery and -33% for PV and inverter), and increased carbon price (10€/ton CO_2_) would result in a good economic return on the PV-battery investments (NPV = 2081€) while the PV-only investment has slightly positive NPV. This shows how a combination of naturally evolving conditions, such as rising electricity prices and falling technology costs, and regulator-imposed incentives, such as self-consumption-only PV subsidies, could create conditions for the deployment of more PV but only coupled with battery energy storage.

The changes of energy tariffs and technology costs will lead to different optimum system sizes. The increase in the imported tariff will stimulate the rising of both PV and battery sizes, while the decrease in the exported tariff can only reduce the size of the PV system but has little effect on the battery sizing. The optimum sizes of both the PV and battery increase as the battery cost declines. However, when the battery cost is lower than 73€/kWh, the increase in PV size becomes less obvious. The decrease in PV cost leads to similar trends of the changes of the optimum system sizes as the battery cost declines, despite the fact that the optimum battery size has little variations when the battery cost is lower than 73€/kWh.

The operating strategy of this PV-battery storage system is to maximize self-consumption, hence storing the excess PV power production in the battery, rather than selling it to the grid, in order to use it later when demand cannot be met by solar energy, thus decreasing the amount of energy bought from the grid. Therefore, it is clear in this context that the battery can add a value to a residential PV system, where the demand is hardly matched by PV generation. In the above-described “optimum” configuration, the PV-only system starts from a 21% self-consumption, leaving a large margin for the battery to increase this value, making the PV-battery investment attractive. On the contrary, an office-type load profile, with a daily-only demand, would be less suitable for PV-battery storage application. Therefore, it can be concluded that such system has a better potential for applications in residential buildings.

As far as energy storage is concerned, there are many other operating strategies. In residential PV-battery storage systems, the operation of the battery can be optimized to achieve an economic optimum [32, 33], such as lowest electricity bill, when variables such as varying electricity tariff are taken into consideration. Another valuable strategy would be maximizing battery life [34, 35] while not compromising too much of the other objectives such as self-consumption. In fact, the battery degradation should be taken into account in more detail in such studies. Despite storage having a great potential in a variety of applications, the authors would like to stress its value in association with renewable energy systems such as PV, either at a residential level or even better at a community level where many PV owners could jointly share the benefits of some energy storage facilities. Overall, self-consumption maximization seems the most natural operating strategy to be followed, and policies, both existing and new, should be pushing towards this direction. Clever operating strategies should be put in place in a smart grid context to optimize the operation of all components while maximizing the overall benefits.

## 4. Methods

### 4.1. System Overview

**Figure 6:**
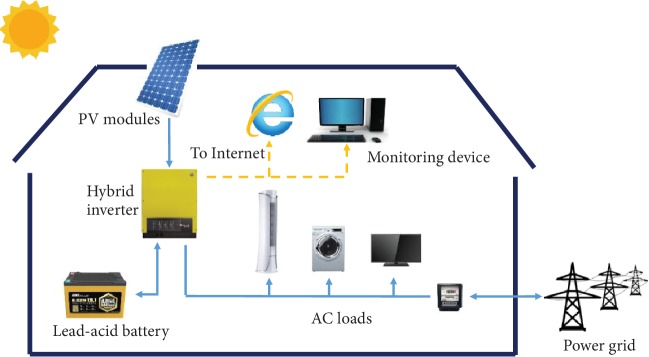
System component overview.

The PV-battery energy storage system is installed in a residential apartment connected to the low-voltage distribution network. The system is composed of PV modules generating renewable energy, advanced lead-acid batteries to store electrical energy, and a hybrid inverter which deals with both DC connections and interfaces on the AC side with loads and grids (see Figure 6).

The authors would like to stress the fact that design and sizing stages have taken place before the start of this work with the idea of providing students and researchers with a system that could be used in different configurations, such as off-grid and on-grid, to carry out a variety of studies on the field of PV-BESS, hence being not optimal to the purposes of this specific work. The details of the subsystems such as the residential apartment, the PV generation system, the battery storage system, and the hybrid inverter can be found in Supplementary Materials ([Supplementary-material supplementary-material-1]).

### 4.2. Modelling of Annual Energy Performance

In the previous section, the real operations of the PV-battery storage system installed at the Shanghai Jiao Tong University Green Energy Lab have been analyzed considering a restricted timescale. However, to properly assess its overall performance, the system should be tested throughout a whole year of operations. In order to achieve this, a computational model has been built using the software TRNSYS and validated with experimental results. The yearly simulations have been run in the actual system configuration and compared to a “PV-only” scenario without batteries. The model is used to simulate other scenarios where some of the fundamental parameters of the systems are changed to assess their effects on the economic viability of such PV-battery systems.

**Figure 7:**
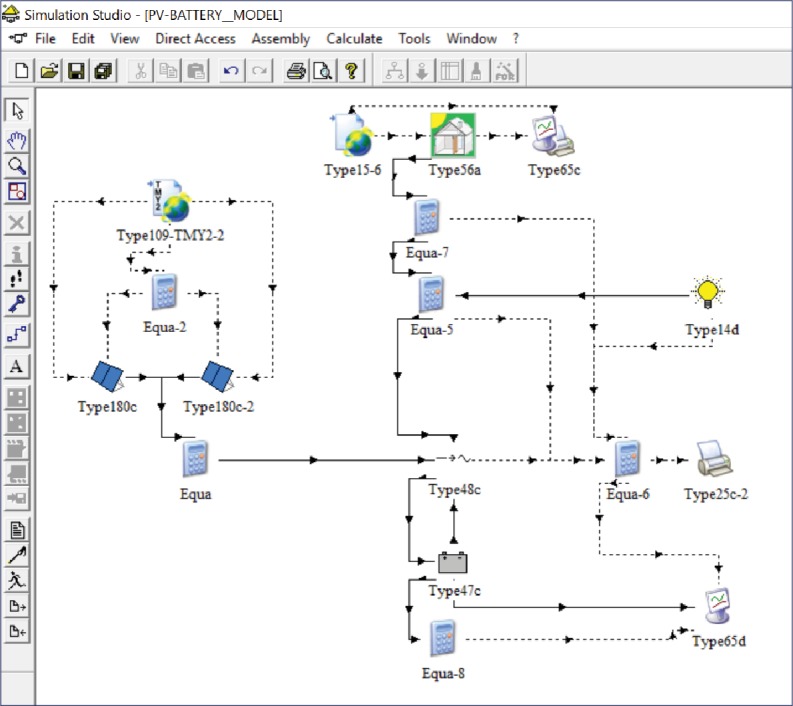
TRNSYS simulation of the residential PV-battery system model.

TRNSYS [36] is a software environment for simulating the behavior of transient systems, especially thermal and electrical energy systems.  Figure 7 shows how this works' model looks like in TRNSYS simulation studio, with all the block connections. It is composed of an electrical model of the PV-battery system and a thermal model of the apartment to calculate heating and cooling thermal loads giving weather data as input. The following description refers to the model structure, while all component data and parameters used in the model are the ones given in the previous section.

The PV system has been modelled by two *Type180c* components, one for the east string and the other for the west string, from the TRNSYS Electrical Library. Type180 is a mathematical model for a photovoltaic generator based on the equivalent circuit of a one-diode model especially intended for PV-arrays consisting of silicon cells. The electrical model used is described in [37]. A dynamic thermal model has also been included [38]. The PV array is assumed to include MPPT [39].

The battery is modelled by *Type47c* from the TRNSYS Electrical Library, a model of lead-acid storage battery operating in conjunction with PV generation. Given the rate of charge and discharge in terms of power, it returns the battery state of charge, voltage, and current over time. It utilizes the Shepherd equations [40], modified by Hyman [41] so as to be more realistic at lower currents; power is given as input.

*Type48c* from the TRNSYS Electrical Library models both the regulator, managing the charging and discharging battery operation on the DC side of the system, and the inverter, converting to AC power from either PV or battery. The battery management system works with the same logic of the real inverter, maximizing self-consumption and minimizing grid withdrawal. It gets the power values from PV (DC side) and load, defining the battery and grid activity according to its logic and through energy balances.

The electrical demand in the model is split into 2 components: HVAC demand and non-HVAC demand. HVAC demand is properly computed through *Type56*, a thermal model of the apartment available from previous works [42]. The model takes in the weather data, HVAC scheduling, internal gains, and other parameters and computes the thermal cooling or heating demand, from which cooling and heating electrical load can be derived applying the heat pump COPs. COPs are assumed to be constant throughout the year, and they are computed from the air-sourced heat pump specifications from which the thermal heating and cooling rates are 11.2 kW and 10 kW, respectively, while the rated electrical input is 3.23 kW and 3.58 kW giving an average COP of 3.47 for heating and 2.79 for cooling. Non-HVAC demand is instead imputed as a daily and weekly routine of assigned power level in *Type14d*.

*Type109* and *Type15* are the weather components feeding data into the PV generation and building thermal load components. The data are in typical meteorological year format, being sets of hourly values for a 1-year period deriving from averaging historic measured data from a large number of years. Simulations were run for a full year with a time step of 5 minutes.

### 4.3. Simulation Model Validation

**Figure 8:**
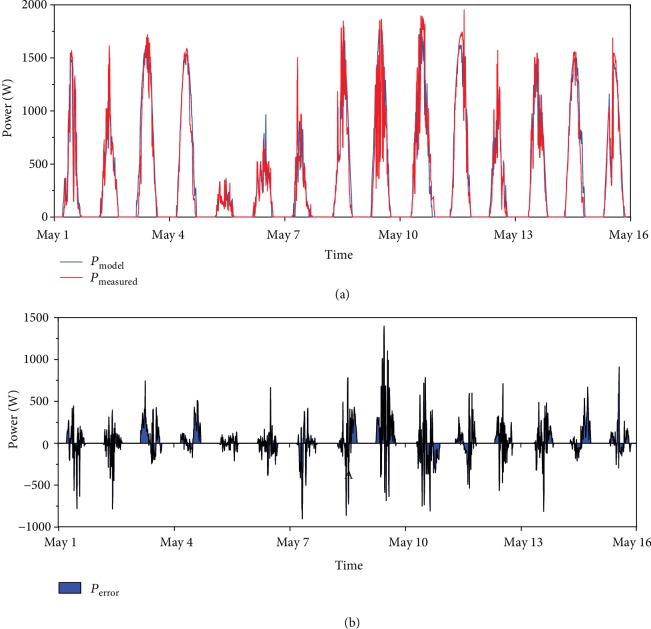
Comparison of modelled and measured PV power output.

The PV model has been validated on a period of 15 days in the first half of the month of May 2018. The measured PV production from the actual system has been compared with the simulated results obtained, imputing the weather data recorded for those days by the university weather station installed at the same building, hence very accurate in terms of location. From Figure 8, the average percentage relative error between the two power curves was high, 23.6%, due to rapid changes of solar radiation caused by cloud cover. However, in terms of cumulative energy, it was coming down to 8.4% and deemed acceptable.

**Figure 9:**
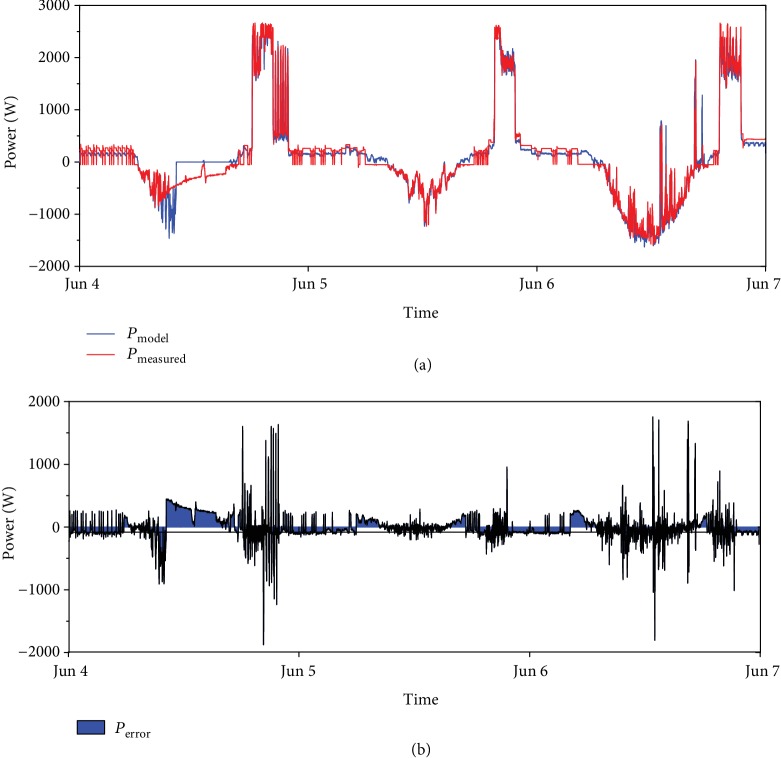
Comparison of modelled and measured battery power.

The inverter and battery components of the model have been validated separately from the PV generation plant, so the measured PV production was used as input, together with the load. The period chosen was 3 days from the 4^th^ to 6^th^ of June 2018. The resulting modelled battery and grid power were checked with measured data. From Figure 9, the model response is satisfying, especially considering that the purpose of this part of the work is not the dynamic assessment of the system operation, but the annual energy flow analysis. In fact, the cumulative energy comparison between the modelled and measured data has resulted fairly accurate even when the power data was showing some model limitations.

Overall, the model response is satisfying, especially considering that the purpose of this part of the work is not the dynamic assessment of the system operation, but the annual energy flow analysis. In fact, the cumulative energy comparison between the modelled and measured data has resulted fairly accurate even when the power data was showing some model limitations. In Section 2, the model is used to simulate a full-year operation both with and without the battery energy storage system.

### 4.4. NPV and Economic Assessment

The main idea of the discounted cash flow analysis is to consider the present value at the time of the start of an investment of all expected inflows and outflows of money throughout the investment period and compare it with a hypothetical similar risk investment. The sum of all those yearly present value flows is the net present value (NPV), which is computed as follows:

(2) $$\begin{array}{c} \text{NPV}=\overset{n}{\underset{j=1}{\sum }}\frac{{\text{NR}}_{j}}{{\left(1+a\right)}^{j}}-\overset{n-1}{\underset{j=0}{\sum }}\frac{I_{j}}{{\left(1+a\right)}^{j}}, \end{array}$$

where *I*_*j*_ is the investment in year *j* and NR_*j*_ is the net revenue obtained in year *j*, calculated from the difference between the gross revenue *R*_*j*_ and maintenance and operation costs *d*_O&M_ as a percentage of the total investment *I*_t_ as follows:

(3) $$\begin{array}{c} {\text{NR}}_{j}=\left(R_{j}-d_{\text{O}\&\text{M}}\right)\cdot I_{t}. \end{array}$$

The most important parameter for a discounted cash flow analysis is the choice of the discount rate (*a*) which could be described as a loan to be repaid or as the expected rate of return from other similar risk investments. In this study, 6% is assumed to be the discount rate value.

Another important factor is to establish the investment length (*n*). In this type of investment, the life expectancy of the plant is considered. However, for PV-battery systems, this is particularly tricky as PV modules have a life expectancy of more than 20 years [43], while lead-acid batteries have much shorter life depending on the type and usage [44]. The manufacturer states that at 70% DOD, the cycle life is 4500. We have limited the discharging process to 60% DOD to be conservative, and by assuming 2 cycles a day, the life expectancy would be a little more than 6 years. Therefore, we would consider an investment timescale of 18 years for the PV-battery system as a whole where the battery is replaced twice at the 7^th^ and 13^th^ year of the investment period (battery pack life is 6 years), while the inverter once at the 10^th^ year (inverter life is 9 years). All components are considered not to have any value after 18 years, including PV, despite having a longer life expectancy. In compensation, no decommissioning costs are considered for any components.

PV module and battery ageing is considered in this NPV analysis by assuming a constant average capacity for both components lower than the nominal one. PV panels are assumed to lose 20% of their peak power output during their life; hence, an average 0.9 coefficient is applied to their output. The most important consequences of battery ageing are capacity and power fade. In this study due to the fact that applied current rates are well under the battery limits (50 Amps of charging and discharging rates is applied, against limits of 150 A and 325 A, respectively), power fade is not considered. Instead, capacity fade is taken into account: the battery is assumed to lose 20% of its usable capacity over its lifetime; this means that 70% DOD of the new battery will be reduced to 50% DOD of the spent one, averaging 60% which is the value used in the experiments.

When it comes to technology cost, the unitary costs of the PV system (monocrystalline type), hybrid inverter, and lead-carbon batteries are 1067 (€/kW), 267 (€/kW), and 220 (€/kWh), coming from the manufacturers. The investment of the PV-battery system can be expressed as

(4) $$\begin{array}{c} I_{\text{t}}=C_{\text{PV}}\cdot S_{\text{PV}}+C_{\text{bat}}\cdot S_{\text{bat}}, \end{array}$$

where *S*_PV_ and *S*_bat_ are the capacities of the PV system and the batteries and *C*_PV_ and *C*_bat_ are the unitary costs of the PV system (with an inverter) and the batteries. The unitary cost for each component comprises balance of system costs, while the maintenance and operation costs (*d*_O&M_) of the PV-battery system in this case are assumed to be 1% of the initial investment.

As for electricity tariffs and PV incentives, the standard electricity tariff in China is a time-of-use tariff. It varies from province to province and is different for the commercial and residential sectors. In Shanghai, the residential tariff is a simple all-year-around tariff composed of the so called “valley” part (low price of 0.041€/kWh at night between 10 p.m. and 6 a.m. and at the following day) and the “peak” part (high price of 0.083€/kWh during the day from 6 a.m. to 10 p.m.) [45]. Taxes and fixed costs are included into those figures. Feed-in tariff (FiT) policy is one of the most popular and effective PV generation incentives, defining a fixed payout per unit electricity generated. In China, the subsidy standard for domestic PV power has remained unchanged at 0.42¥/kWh (equal to 0.056€/kWh) since 2013 [46]. Furthermore, in addition to state subsidies, there are local subsidies. In Shanghai, the local subsidy amounts to 0.40¥/kWh for residential and 0.25¥/kWh for commercial (equal to 0.053€/kWh and 0.033€/kWh, respectively). State subsidies have a 20-year validity while local subsidies have a 5-year validity [47]. The export tariff for excess PV generation sold to the grid amounts to 0.405¥/kWh, equal to 0.054€/kWh. Instead, subsidy policies for energy storage in China are lacking compared to those for PV power, with no subsidy or tax relief being in place. The gross revenue can be expressed as

(5) $$\begin{array}{c} R_{j}=C_{\text{inc}}\cdot E_{\text{PV}}+C_{\text{export}}\cdot E_{\text{export}}-C_{\text{import}}\cdot E_{\text{import}}+C_{\text{import}}\cdot E_{\text{load}}, \end{array}$$

where *C*_inc_, *C*_export_, and *C*_import_ are the tariffs for incentives, exported electricity, and imported electricity, respectively, and *E*_PV_, *E*_export_, *E*_import_, and *E*_load_ are the amounts of PV-generated, exported, imported, and load electricity. *E*_export_ and *E*_import_ are the functions of SSR (self-sufficiency ratio), SCR (self-consumption ratio), *E*_PV_, and *E*_load_:

(6) $$\begin{array}{c} E_{\text{export}}=E_{\text{PV}}\cdot \left(1-\text{SCR}\right), \end{array}$$

(7) $$\begin{array}{c} E_{\text{import}}=E_{\text{load}}\cdot \left(1-\text{SSR}\right). \end{array}$$

Based on equations (2), (3), (4), (5), (6), and (7), the equation of NPV can be expressed as follows and it is clear to see how different factors affect NPV:

(8) $$\begin{array}{c} \text{NPV}=\left[\overset{n}{\underset{j=1}{\sum }}\frac{1}{{\left(1+a\right)}^{j}}\right]\cdot \left[C_{\text{inc}}E_{\text{PV}}+C_{\text{export}}E_{\text{PV}}\left(1-\text{SCR}\right)+C_{\text{import}}E_{\text{load}}\text{SF}\right]-\left[1+\overset{n}{\underset{j=1}{\sum }}\frac{d_{\text{O}\&\text{M}}}{{\left(1+a\right)}^{j}}\right]\cdot \left(C_{\text{PV}}\cdot S_{\text{PV}}+C_{\text{bat}}\cdot S_{\text{bat}}\right). \end{array}$$

It is worth noting that the paper discusses the financial viability of residential PV-battery systems and that the results may differ in different technology cost and energy policy settings. In this paper, we use the real costs from the equipment manufacturers and energy tariffs in China. The literature review (see Table 11) shows that a plenty of studies have been conducted in European countries while cases in China are hardly seen. In addition, the cost and tariff settings in this paper do not deviate much from the cases in Europe, implying the reasonability of the assumption in this paper.

## Figures and Tables

**Table 1:** Non-HVAC electrical load daily composition.

Type of electrical load	Value (W)	Daily schedule (h)
Base load	150	0-24
Lights	400	Morning (7:00-8:00)-evening (18:00-23:00)
TV/laptop	300	Evening (19:00-22:00)
Induction cooker	2000	Evening (18:30-19:00)

**Table 2:** Simulation results for systems with actual and optimum sizes.

Parameters	Actual (residential, SH)		Optimum (residential, SH)		Optimum (nonresidential, SH)		Optimum (residential, HK)	
	PV+bat.	PV only	PV+bat.	PV only	PV+bat.	PV only	PV+bat.	PV only
Battery sizes	24 kWh	No	8 kWh	No	8 kWh	No	8 kWh	No
PV sizes	3 kW	3 kW	3.5 kW	3.5 kW	3.5 kW	3.5 kW	3.5 kW	3.5 kW

Battery charging (kWh)	2617	0	1933	0	1588	0	1830	0
Battery discharging (kWh)	2125	0	1478	0	1273	0	1391	0
Demand (kWh)	4322	4322	4322	4322	4432	4432	4438	4438
Grid import (kWh)	1396	3436	1994	3413	1597	2803	2229	3557
Grid export (kWh)	184	2801	1465	3399	1077	2716	1107	2967
PV production (kWh)	3742	3742	4344	4344	4344	4344	3848	3848
Self-consumed energy (kWh)	2926	886	2328	908	2834	1628	2209	881
Self-consumption rate	79%	24%	54%	21%	65%	37%	57%	23%
Self-sufficiency rate	68%	20%	54%	21%	64%	37%	50%	20%
Grid electricity cost (€)	85	233	116	231	91	171	137	246
PV incentive earning (€) (1-5 years)	417	417	487	487	487	487	431	431
PV incentive earning (€) (6-20 years)	209	209	243	243	243	243	215	215
Electricity sold earning (€)	10	151	79	184	58	147	60	160
Base electricity bill (€) (no PV, no battery)	306	306	306	306	306	306	319	319
NVP for 18 years (€)	-10357	1392	-2467	1715	-2382	2008	-3137	1072

**Table 3:** Combined import and export tariff sensitivity analysis.

Scenario	Import tariff (€/kWh)	Export tariff (€/kWh)
1	0.083	0.054
2	0.124 (1.5×)	0.041 (0.75×)
3	0.166 (2×)	0.027 (0.5×)
4	0.208 (2.5×)	0.013 (0.25×)
5	0.249 (3×)	0

**Table 4:** NPV trend in a decreasing PV generation subsidy context.

	State subsidy (20 years) (€/kWh)	Local subsidy (5 years) (€/kWh)	NPV of PV-battery (€)	NPV of PV only (€)
1	0.056	0.053	-2467	1715
2	0.056	0	-3443	739
3	0.042 (0.75×)	0	-4102	80
4	0.028 (0.5×)	0	-4761	-578
5	0.014 (0.25×)	0	-5419	-1237
6	0	0	-6078	-1896

**Table 5:** NPV trend with increasing self-consumption-only PV generation incentives.

	Self-consumption State subsidy (20 years) (€/kWh)	Self-consumption Local subsidy (5 years) (€/kWh)	NPV of PV-battery (€)	NPV of PV only (€)
1	0.056	0.053	-3685	-1109
2	0.084 (1.5×)	0.08 (1.5×)	-2488	-716
3	0.112 (2×)	0.106 (2×)	-1292	-323
4	0.140 (2.5×)	0.133 (2.5×)	-95	70
5	0.168 (3×)	0.159 (3×)	1101	463

**Table 6:** Reduction rate of battery and PV cost.

Term	Battery cost reduction	NPV of PV-battery (only battery cost reduction) (€)	PV and inverter cost reduction	NPV of PV-battery (PV, battery, and inverter cost reduction) (€)	NPV of PV only (PV, battery, and inverter cost reduction) (€)
—	0%	-2467	0%	-2467	1715
Short	50%	-586	17%	797	2830
Medium	67%	54	33%	2326	3627
Long	80%	543	44%	3383	4126

**Table 7:** NPV trend with increasing carbon price.

Term	Carbon price (€/ton CO_2_)	NPV of PV-battery (€)	NPV of PV only (€)
—	0	-2467	1715
Short	5	-2170	2012
Medium	10	-1873	2309
Long	20	-1278	2904

**Table 8:** Possible future scenarios.

Parameters	Current	Mild	Medium	Strong
Import tariff (€/kWh)	0.083 (peak)	0.124 (1.5×)	0.166 (2×)	0.208 (2.5×)
	0.041 (valley)	Flat tariff		
Export tariff (€/kWh)	0.054	0.027 (0.5×)	0.013 (0.25×)	0
PV generation subsidy (€/kWh)	0.056 (20 years)	0.028 (0.5×) (20 years)	None	None
	0.053 (5 years)	0.026 (0.5×) (5 years)		
PV self-consumption subsidy (€/kWh)	None	None	0.056 (20 years)	0.112 (2×)
			0.053 (5 years)	0.106 (2×)
Battery cost (€/kWh)	220	-50%	-66%	-80%
Hybrid inverter cost (€/kW)	267	-17%	-33%	-44%
PV cost (€/kW)	1067	-17%	-33%	-44%
Carbon price (€/ton CO_2_)	0	5	10	20

**Table 9:** Optimum sizes of the PV system and batteries and the corresponding NPV (kW/kWh/€) for different energy tariffs.

Import	Export			
	0.054 (€/kWh)	0.036 (€/kWh)	0.018 (€/kWh)	0 (€/kWh)
0.083 (€/kWh)	0/0/0	0/0/0	0/0/0	0/0/0
0.286 (€/kWh)	1.4/1/772	1.2/1/675	1/1/604	0.9/1/550
0.500 (€/kWh)	6/12/4289	5/11/3836	4.4/11/3498	3.6/9/3205
0.704 (€/kWh)	7.9/17/12716	7/16/11970	6.6/17/11347	5.9/16/10842

The data are presented as PV system optimum size/battery optimum size/NPV.

**Table 10:** Optimum sizes of the PV system and batteries and the corresponding NPV (kW/kWh/€) for different technology costs.

PV cost	Bat. cost			
	220 (€/kWh)	110 (€/kWh)	73 (€/kWh)	44 (€/kWh)
1334 (€/kW)	1.4/1/772	1.7/2/1105	4.5/14/2040	5.3/21/3505
1107 (€/kW)	1.9/1/1129	4.6/10/1781	6/16/3213	6.6/23/4864
880 (€/kW)	3.4/1/1683	8/13/3159	8.7/18/4823	9/23/6620
747 (€/kW)	9.9/1/2377	10/13/4406	10/18/6135	10/22/7926

**Table 11:** The comparison of energy policies and technology costs in different literatures.

Literature	Location	Year	PV price (/kWp) (with an inverter)	Bat. price (/kWh)	Export (/kWh)	Import (/kWh)	Incentive (/kWh)	Profitability
This paper	China	2019	1334€	220€ (lead-carbon)	0.054€	0.083€	0.056€	No
[18]	Germany	2014	1600€	500€ (Li-ion)	0.035€	0.295€	—	Yes
[19]	Italy	2016	2000€	171€ (lead-acid)	0.14€	0.19€	—	No
[20]	Italy	2018	Not provided	600€ (Li-ion)	0.11€	0.165€	—	No
[21]	Portugal	2017	1605€	462€ (Li-ion)	0.042€	0.19€	—	No
[22]	Germany	2014	1700€	171€ (lead-acid)	0.042€	0.288€	—	Yes
[23]	The UK	2017	1547€	1134€ (Li-ion)	0.044€	0.112€	0.05€	No
[24]	The UK	2017	Not provided	557€ (Li-ion)	0.054€	0.153€	0.149€	No
[25]	Germany	2014	1000€	600€ (Li-ion)	0.02€	0.34€	—	Yes

## Supplementary Materials

Supplementary Materials Figure S1: residential apartment on the second floor of the Green Energy Laboratory. Figure S2: PV generation plant. Figure S3: (from left to right) single battery, battery pack with an inverter and battery housing. Figure S4: grid-connected DC-coupled PV-BESS. Table S1: list of the electrical demand items. Table S2: PV module parameters. Table S3: single battery specifications. Table S4: inverter specifications.
