# Supplementary material for: Solar PV-Battery-Electric Grid-Based Energy System for Residential Applications: System Configuration and Viability
Source: Research (Wash D C). 2019 Oct 8;2019:3838603. doi: 10.34133/2019/3838603 (PMC6946270; doi:10.34133/2019/3838603)
Supplement: Supplementary Materials — Figure S1: residential apartment on the second floor of the Green Energy Laboratory. Figure S2: PV generation plant. Figure S3: (from left to right) single battery, battery pack with an inverter and battery housing. Figure S4: grid-connected DC-coupled PV-BESS. Table S1: list of the electrical demand items. Table S2: PV module parameters. Table S3: single battery specifications. Table S4: inverter specifications. [file 3838603.f1.docx]

#### 1 Residential apartment

The system electricity demand comes from a residential apartment which is located on the second floor of GEL, Figure S1. It is a fully functional flat with a living room, a kitchen, a small study room, a double bedroom and a bathroom.


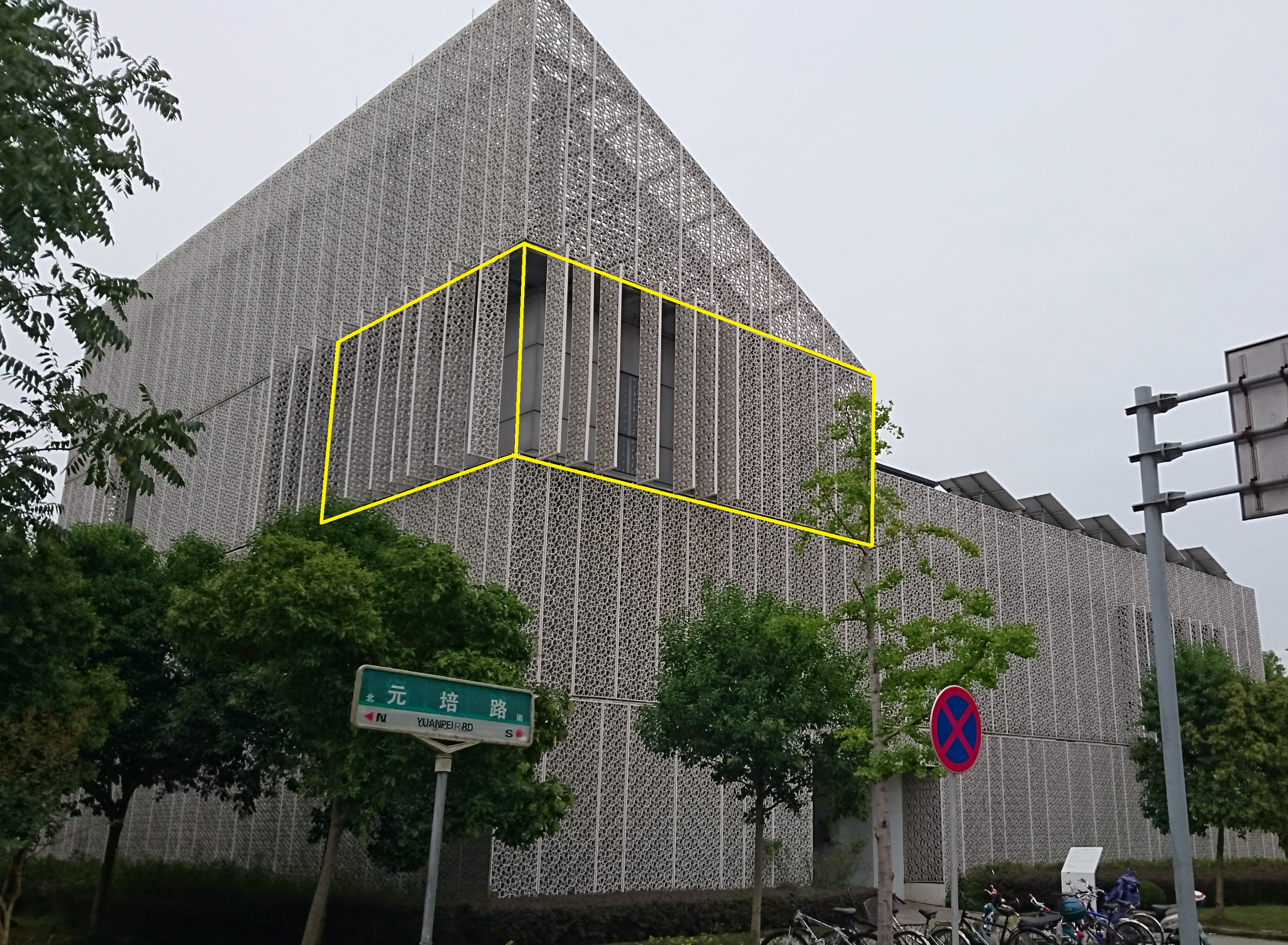

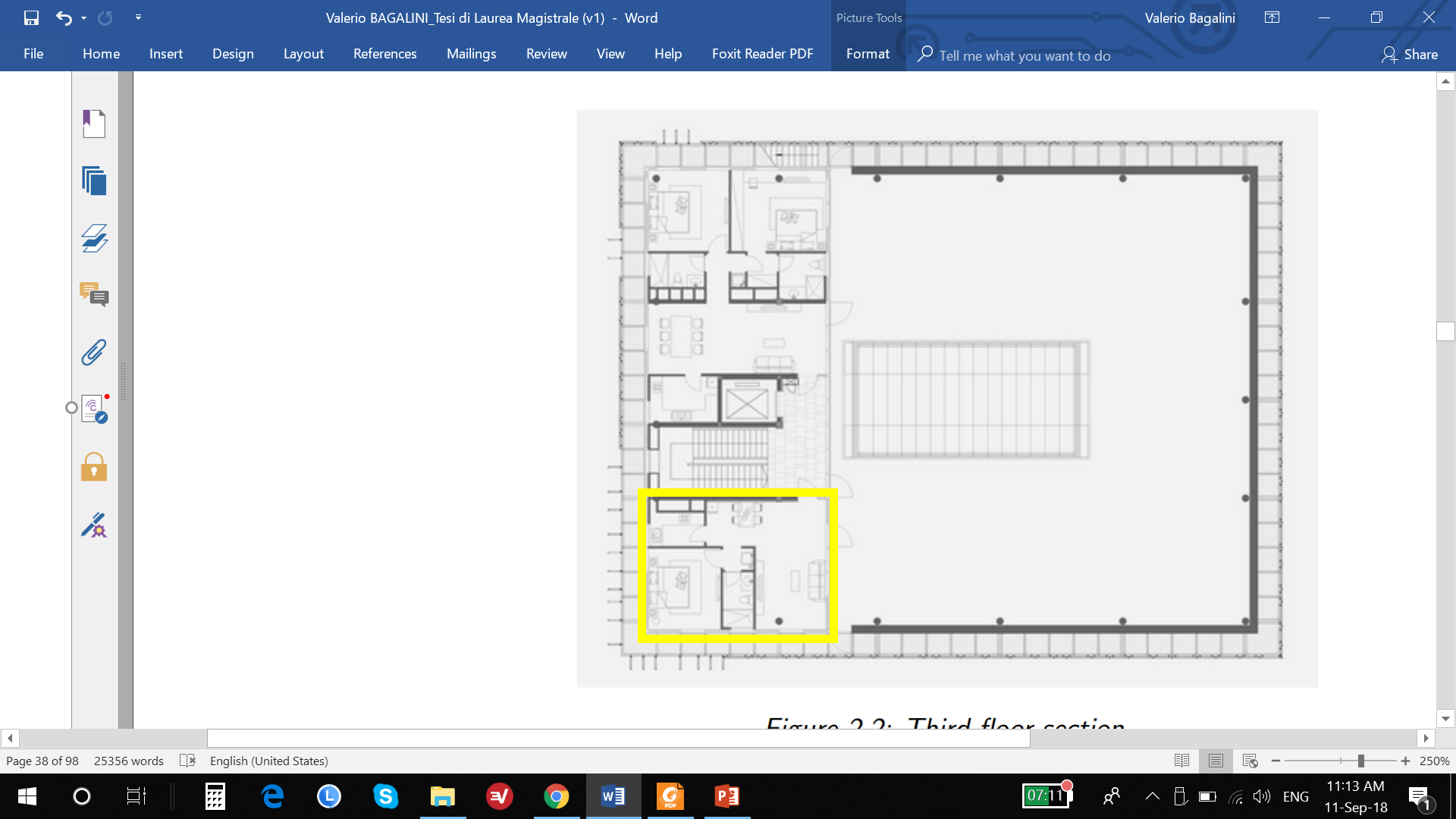


Figure S1: Residential apartment on the second floor of the Green Energy Laboratory

The apartment has an area of 60 m^2^, a length of 9 m, a width of 6.6 m and a height of 3 m. The south facade, west facade and north facade are external walls. East facade and ground are adjacent to other laboratories. The data of the building and the thermal model built using it are taken from the previous study (*Bo, W., Jingyi, W., & Ruzhu, W. (2013). Performance Analysis of Energy-efficient Apartment in Hot Summer and Cold Winter Zone (in Chinese). Electricity and Energy (Vol. 34, No. 2), 124-127.*) on this apartment on building energy efficiency. The apartment is equipped with energy efficient home appliances, such as induction cooker, refrigerator, washing machine, dishwasher and energy saving lighting. An air-sourced heat pump is used for both air conditioning operations of winter heating and summer cooling. A list of the electrical demand items is provided by Table S1.

Table S1: List of the electrical demand items

| List of electrical demand items | Nominal electrical power |
| --- | --- |
| Air Sourced Heat Pump for Air Conditioning | 3.23/3.58 kW (heating/cooling) |
| Induction cooker | 2.6 kW |
| Range hood | 180 W |
| Refrigerator | 0.65kWh/24 hours |
| TV(2 sets) | 275 & 300 W |
| Other cooking appliances | 1.2 kW |
| Lighting | 800W |

The air-source heat pump is composed by one external unit and two internal units. The outdoor unit is single phase/220 V/50 Hz machine. The thermal heating and cooling rates are 11.2 kW and 10kW respectively, while the rated electrical input is 3.23 kW and 3.58kW giving an average COP of 3.47 for heating and 2.79 for cooling. It has a variable speed motor to modulate the output, rather than having an ON/OFF control strategy. After the air-conditioning, the second most demanding electrical equipment is the induction cooker. It will be shown later how it affects the electricity demand especially in terms of power.

The house is designed to be all-electric, without gas burning equipment. Hot water in fact is produced by another dedicated air source heat pump water heater with 150 Litre water tank. This facility however was not available during our experiments, hence was excluded by our scope. Another unavailable equipment was the Heat Recovery Ventilation (HRV) unit, therefore fresh air was assumed to be directly infiltrating from the outside (0.5 house volume per hour is assumed in the computational model), with loss of energy saving potential.

#### 2 PV generation system

The PV system is conveniently located on top of the roof of the glass conservatory on the south-facing side of the apartment (Figure S2). It is composed by 30 mono-crystalline silicon PV panels, each of them has 100 W of rated peak power, totaling 3 kW peak. The panels are arranged on top of the 20 degree inclined roof, divided into 2 strings of 15 panels in series one facing west, the other facing east. The module parameters are shown in Table S2.


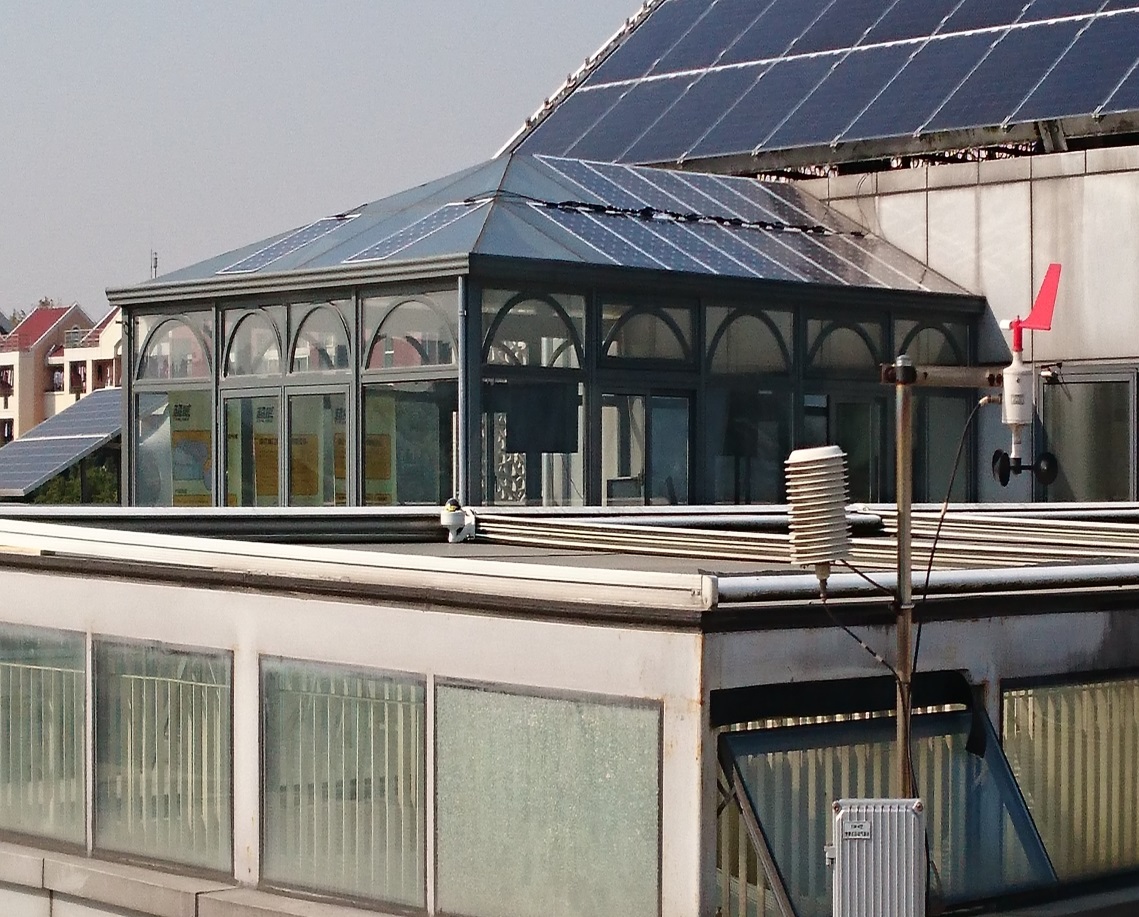


Figure S2: PV generation plant

Table S2: PV module parameters

| Parameters | Values |
| --- | --- |
| Peak power (Pmax) | 100 W |
| Peak voltage (Vmp) | 18V |
| Open circuit voltage (Voc) | 21.6 V |
| Peak current (Imp) | 5.55 A |
| Short circuit current (Isc) | 6.06 A |
| Solar panel working temperature range | -40/85 ^o^C |

#### 3 Battery Energy Storage System

The advanced lead-acid batteries are AGM (absorbent glass mat) VRLA (valve regulated lead acid) type, with carbon added to the negative discharging plate to improve current rates and life performance, they are known as LEAD-CARBON batteries. In the energy system, each individual battery (see Figure S3) is a big single cell outputting 2V with a big capacity of 500Ah, making it a 1kWh cell. 24 batteries are connected in series giving the pack 48V and 24kWh of overall capacity.


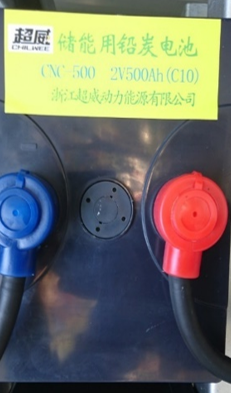

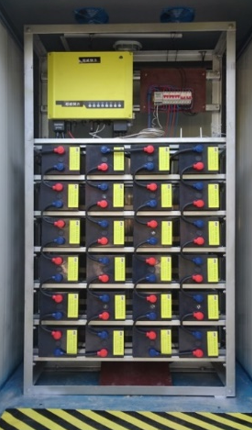

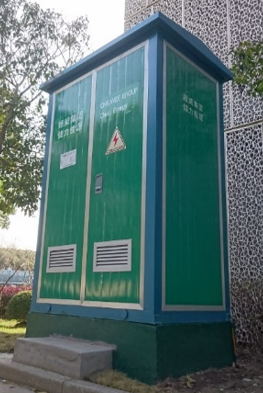


Figure S3: (from LEFT to RIGHT) Single battery, battery pack with inverter and battery housing

Life of lead-acid batteries is shortened by high DOD cycling and high current rates, especially during charging. However due to its advanced design, for this lead-carbon battery the manufacturer suggests a DOD limit of 70% to maintain its expected life of 4600 cycles. To be conservative, a 60% DOD limit has been self-imposed, reducing the usable battery capacity from 24kWh (nominal battery capacity) down to 14.4kWh. Main specifications are shown in Table S3.

Table S3: Single battery specifications

| Parameters | Values |
| --- | --- |
| Rated voltage | 2 V |
| Rated Energy Capacity | 1000 Wh (down to 1.8 V) |
| Dimension | 193 mm×175mm×542mm |
| Weight | 42 kg |
| Single charge voltage (equal charge/float charge) | 2.32 V/2.23 V |
| Maximum charging power (current) | 300 W (150 A) |
| Maximum discharge power (current) | 570 W (325 A) |
| Self-discharge rate | ≤ 2%/month (25 ^o^C) |
| Best use temperature | 20 ~ 30 ^o^C |

#### 4 Hybrid inverter

In this work, the HYBRID bi-directional inverter (see Table S4) connects on the DC side including both PV system and the batteries (DC coupled), shown as Figure S4. The two PV arrays are connected separately to the inverter, which comprises MPPT (maximum peak power tracking) systems (which is another DC/DC converter) for each string. A charger/controller manages the batteries. AC output comprises a limited backup output for basic load, and the main output which is directed to the load/grid connection. A separate electrical panel is necessary to tee-off the load connection from the grid inverter connection. This topology allows the inverter to be completely independent from the grid-load route, the amount of load coming from the grid is therefore not limited by the presence of the PV-battery system. Beside the energy system elements, with the inverter package a communication system is included, comprising meters and communication lines gathering a good deal of data and allowing for remote monitoring and control.


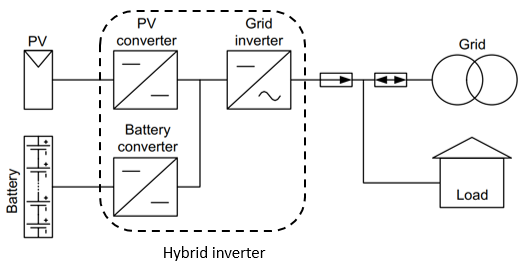


Figure S4: Grid connected DC coupled PV-BESS

Table S4: Inverter specifications

|  | Parameters | Values |
| --- | --- | --- |
| Battery pack | Battery type | Lead-acid |
|  | Number of units in series | 24 |
|  | Nominal battery pack voltage | 48 V |
|  | Total battery pack capacity | 24 kWh |
|  | Usable capacity (60% DOD) | 14.4 kWh |
|  | Max. Charging & discharging power | 3600 W |
| PV string | Max. DC input power | 4600 W |
|  | No. of MPPTs trackers | 2 |
|  | No. of string per MPPTs tracker | 1 |
| AC output | Max. apparent power output to grids | 3680 VA |
|  | Max. apparent power from grids | 7360 VA |
|  | Max. apparent power output (backup) | 3680 VA |
| Efficiency | Max. battery to load efficiency | 94% |
|  | MPPT efficiency | 99.9% |

The most important feature of the inverter is its capability to manage the energy flows, as it is equipped with a built-in Energy Management System. This work is carried out considering the “General mode”, which maximizes self-consumption from a static energetic point of view, without any consideration on the economic side (such as considering the varying electricity tariffs) or on the dynamic energy consumption side. PV generated energy firstly meet the local demand. In case of excess energy, it charges the battery and only then is sold to the grid. Battery will only charge with excess solar power, not by grid. When PV power is lower than load, battery will discharge. If PV and battery do not supply enough power to meet demand, the Grid supplies the difference.
